# Reactions in Electrodeposited Cu/Sn and Cu/Ni/Sn Nanoscale Multilayers for Interconnects

**DOI:** 10.3390/ma9060430

**Published:** 2016-05-31

**Authors:** Pay Ying Chia, A. S. M. A. Haseeb, Samjid Hassan Mannan

**Affiliations:** 1 Department of Mechanical Engineering, Faculty of Engineering, University of Malaya, Kuala Lumpur 50603, Malaysia; chiapayying@siswa.um.edu.my; 2 Physics Department, King’s College London, Strand, London WC2R 2LS, UK; samjid.mannan@kcl.ac.uk

**Keywords:** thin films, electrodeposition, liquid-solid reactions, solid state reactions, diffusion, intermetallic compounds

## Abstract

Miniaturization of electronic devices has led to the development of 3D IC packages which require ultra-small-scale interconnections. Such small interconnects can be completely converted into Cu-Sn based intermetallic compounds (IMCs) after reflow. In an effort to improve IMC based interconnects, an attempt is made to add Ni to Cu-Sn-based IMCs. Multilayer interconnects consisting of stacks of Cu/Sn/Cu/Sn/Cu or Cu/Ni/Sn/Ni/Sn/Cu/Ni/Sn/Ni/Cu with Ni = 35 nm, 70 nm, and 150 nm were electrodeposited sequentially using copper pyrophosphate, tin methanesulfonic, and nickel Watts baths, respectively. These multilayer interconnects were investigated under room temperature aging conditions and for solid-liquid reactions, where the samples were subjected to 250 °C reflow for 60 s and also 300 °C for 3600 s. The progress of the reaction in the multilayers was monitored by using X-ray Diffraction, Scanning Electron Microscope, and Energy dispersive X-ray Spectroscopy. FIB-milled samples were also prepared for investigation under room temperature aging conditions. Results show that by inserting a 70 nanometres thick Ni layer between copper and tin, premature reaction between Cu and Sn at room temperature can be avoided. During short reflow, the addition of Ni suppresses formation of Cu_3_Sn IMC. With increasing Ni thickness, Cu consumption is decreased and Ni starts acting as a barrier layer. On the other hand, during long reflow, two types of IMC were found in the Cu/Ni/Sn samples which are the (Cu,Ni)_6_Sn_5_ and (Cu,Ni)_3_Sn, respectively. Details of the reaction sequence and mechanisms are discussed.

## 1. Introduction

As the electronic industry is moving towards miniaturization, three-dimensional (3-D) IC packages are being aggressively pursued. In 3-D packages, IC chips are stacked on top of each other to save space and to improve performance. In miniaturized packages, through-Si-vias (TSVs) and solder micro-bumps are used as interconnects, the size of which ranges from a few micrometres to about 50 micrometres [1,2]. In such small interconnects, Cu metallization and Sn-rich solder alloys react and transform completely into Cu-Sn intermetallic compounds (IMC), e.g., Cu_6_Sn_5_ and Cu_3_Sn after reflow. Intermetallic compounds are generally known to be brittle and can adversely affect the reliability of the interconnection particularly in applications involving shock load. Therefore, there is a need to investigate ways to improve the properties of Cu-Sn IMC.

One way to address this could be to add a third element to Cu-Sn based IMCs. Out of different metallic elements studied, Ni is suggested to be a good candidate for addition into solders [3,4,5,6]. Studies have shown that Ni can suppress *ε*-Cu_3_Sn even when added at a small concentration [3,4,5]. Cu_3_Sn is considered to be more detrimental to reliability [7]. Furthermore, Ni shows marked solubility in *η*-Cu_6_Sn_5_ IMC and can influence its properties. Nogita and Nishimura [6] reported that with the addition of 9% Ni to the (Cu,Ni)_6_Sn_5_ IMC, polymorphic phase transformation of the Cu-Sn IMC from the hexagonal structure to the monoclinic structure can be prevented. Cu-Ni-Sn IMC has been reported to have higher hardness and Young’s modulus [8,9,10,11].

Addition of Ni to interconnects has been done in the form of nanoparticles in the recent past. It has been shown [3] that Ni nanoparticles of ~20 nm size undergoes reactive dissolution during reflow leading to in-situ alloying. It is, therefore, expected that if thin nanometric layers of Ni is introduced in Cu/Sn based interconnects in the form of multilayers, the former can get intermixed with Cu and Sn resulting in Ni alloyed IMC. This work investigates the formation of multilayered interconnects containing layers of Cu, Ni and Sn by electrodeposition. The samples are then subjected to intermixing under different conditions.

Considerable amount of work has been done in the past on the Cu/Sn system, both for solid state reactions and solid-liquid reactions. During solid state reactions, it has been found that Cu_6_Sn_5_ IMC forms at room temperature. One study reported that it can form at −2 °C [12]. With the use of Auger electron spectroscopy and W markers, it was found revealed that Cu is the main diffusing species during the formation of *η*-Cu_6_Sn_5_ at room temperature. Chopra and co-workers suggested that at the initial stage, the formation of *η*-Cu_6_Sn_5_ took place in the Sn matrix in the form of precipitates through rapid diffusion of Cu into bulk Sn [13]. However, a recent study by Sobiech and co-workers showed that after five days of room temperature aging, Cu atoms diffuse preferentially along Sn grain boundaries where the *η*-Cu_6_Sn_5_ phase formed first [14]. As has been found in the literature, Cu and Sn can mix readily at room temperature. This can lead to premature formation of IMC even before reflow. This may result in difficulties in obtaining a good interconnection, as Cu and Sn can be consumed completely prior to joint formation, particularly when the interconnections are small as in the case of 3D packages. The addition of the nanometric Ni layers between Cu and Sn as proposed above can help to prevent the premature intermixing before reflow. Ni layers should be thin enough though to undergo complete dissolution during reflow. Little information is available in the literature on the intermixing behaviour in Cu/Ni/Sn multilayered system.

In this work, we introduced ultrathin Ni layers of thickness 35 nm to 150 nm between Cu and Sn layers. The motivation is to prevent premature reaction between Cu and Sn prior to reflow. Another motivation is to form, during reflow, Cu-Sn IMCs alloyed with Ni which are expected to lead to better mechanical properties and hence improved reliability of interconnects. Cu/Sn and Cu/Ni/Sn multilayers system were prepared by electrodeposition. Both systems were investigated in terms of IMC formation after 1 day and 24 days of room temperature aging. The effect of Ni in the Cu/Sn system is also studied after both short and long reflow to investigate solid-liquid state reactions.

## 2. Results

### 2.1. Solid State Reactions of Cu/Sn and Cu/Ni/Sn Multilayers

**Figure 1:**
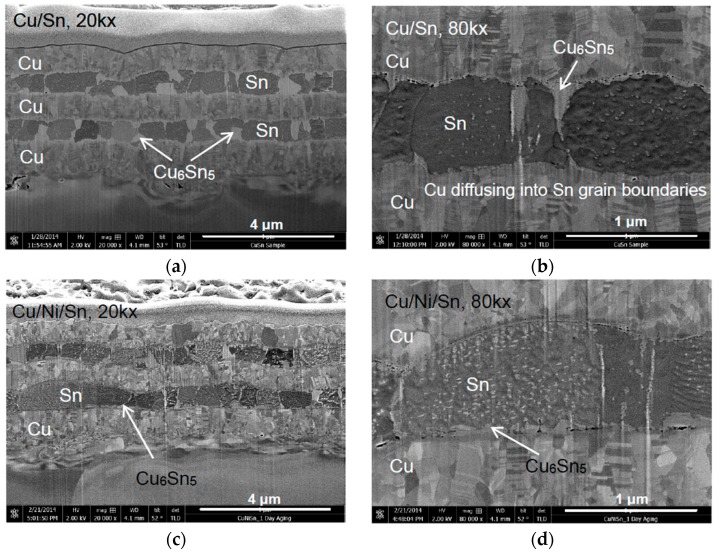
Field Emission Scanning Electron Microscope (FESEM) micrographs showing FIB milled cross sectional images of Cu/Sn (**a**,**b**) and Cu/Ni/Sn (**c**,**d**) aged at room temperature for one day at 20,000× and 80,000× magnifications, respectively.

**Figure 2:**
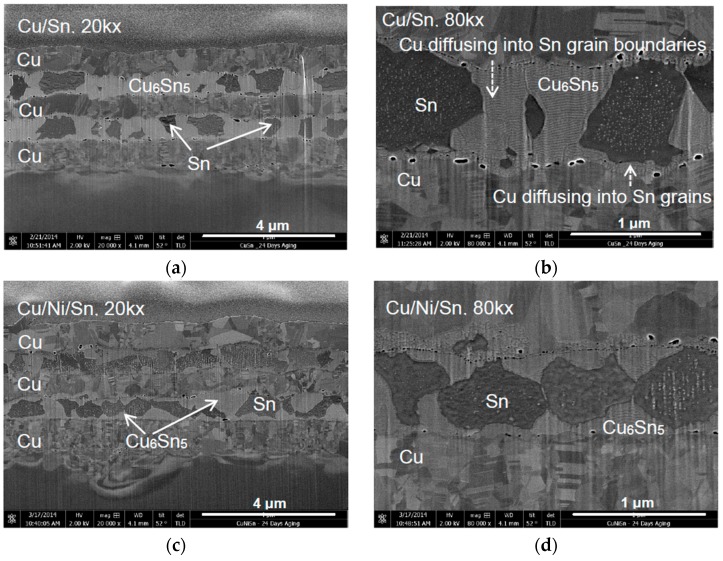
FESEM micrographs showing FIB milled cross sectional images of Cu/Sn (**a**,**b**) and Cu/Ni/Sn (**c**,**d**) aged at room temperature for 24 days at 20,000× and 80,000× magnifications, respectively.

Figure 1 and Figure 2 show FESEM micrographs of FIB milled Cu/Sn and Cu/Ni/Sn samples after room temperature aging for one day and 24 days, respectively. The Cu/Ni/Sn samples used for solid state reactions contain Ni layers with 70 nm thickness. Layers with darker contrast are Sn while that with lighter contrast are Cu (Figure 1a,c). Micrographs at higher magnification (Figure 1b,d) show that the Cu layer contains fine columnar grains with axis perpendicular to the interface. The width of the columnar Cu grain is about 100–200 nm. Each columnar grain contains nanotwins with a twinning plane parallel to the interface. Sn layers (Figure 1b,d) contain more or less equiaxed grains with lateral width of about 1–2 μm. Within the dark grey tin layers, a phase with a lighter grey shade is seen, which corresponds to Cu_6_Sn_5_ IMC (Figure 1b).

After the Cu/Sn sample was aged for one day, Cu_6_Sn_5_ is seen as thin columns along the Sn grain boundaries (Figure 1a,b). Nanometric voids can be seen between Cu and Cu_6_Sn_5_ IMCs layers. In the Cu/Ni/Sn system, IMC was seen to form at the Ni/Sn interface and also along Sn grain boundaries after one day of room temperature aging (Figure 1c,d). However, the extent of IMC formation in the Cu/Ni/Sn sample was smaller when compared to that of the Cu/Sn sample.

After 24 days of room temperature aging, it is observed (Figure 2a) that in the Cu/Sn system, most of the Sn has reacted with Cu to form wider grains of Cu_6_Sn_5_ IMC. Some of these grains have merged with neighbouring grains forming blocks of Cu_6_Sn_5_ IMC separated from each other by some unreacted Sn. Additionally, a layer of Cu_6_Sn_5_ IMC is seen at the Cu/Sn interface (Figure 2a,c). From these observations, it is suggested that Cu_6_Sn_5_ IMC grew simultaneously in two directions, depicted by dashed arrow in Figure 2b: (i) it grew along the Sn grain boundary and (ii) grew into bulk Sn parallel to the Cu/Sn interface. Furthermore, voids formed in between Cu and Cu_6_Sn_5_ IMC have grown in size (Figure 2b) as compared to those after one day of aging (Figure 1b). In the Cu/Ni/Sn system, it is observed that the extent of formation of IMC is much less. There is still a significant amount of unreacted Sn present in this sample. However, the extent of the growth along the grain boundaries is less in the Cu/Ni/Sn sample as compared with that of the Cu/Sn sample. The diffusion of Cu into Sn through the grain boundary is thought to have been impeded by Ni atoms that may have segregated to the grain boundaries (Figure 2d).

Table 1 shows the number of voids for each range of the void’s diameter for both Cu/Sn and Cu/Ni/Sn samples aged at room temperature. The number of voids was calculated from four micrographs for each condition.

After one day of room temperature aging, the Cu/Sn sample exhibits an average void number of 12 per micrometre along the interface, while the average void number in the Cu/Ni/Sn sample is 11 per micrometre. After 24 days of aging, the average number of voids in the Cu/Sn sample increased to 13.2 per micrometre. However, the number of voids with diameter smaller than 20 nm is found to be 172. In the Cu/Ni/Sn system, the average number of voids is 14.8 per micrometre with the number of voids with diameter smaller than 20 nm is 202. Though there is not much difference in the number of voids formed, fewer voids with larger diameter (voids with diameter bigger than 20 nm) are seen to have formed in the Cu/Ni/Sn sample (Figure 2). The number of voids with diameter smaller than 20 nm is more in the Cu/Ni/Sn sample compared with that in Cu/Sn sample after 24 days of room temperature aging.

### 2.2. Solid-Liquid State Reactions of Cu/Sn and Cu/Ni/Sn Multilayers

#### 2.2.1. Short Reflow

**Figure 3:**
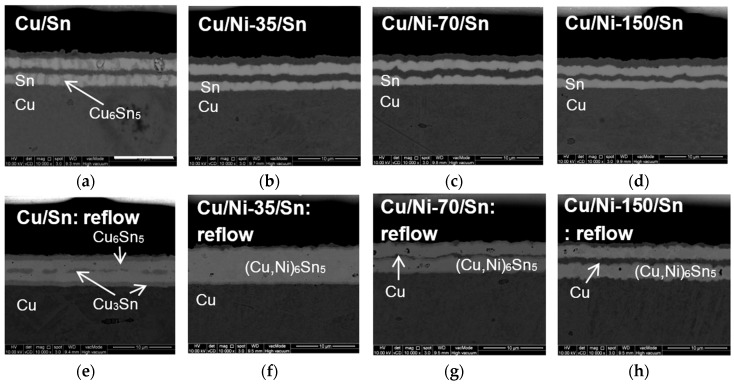
FESEM micrographs showing cross sectional images of (**a**,**e**) Cu/Sn; (**b**,**f**) Cu/Ni-35/Ni; (**c**,**g**) Cu/Ni-70/Ni; and (**d**,**h**) Cu/Ni-150/Ni before and after reflow at 250 °C for 60 s, respectively, at a magnification of 10,000×.

Figure 3 shows the micrographs for Cu/Sn and Cu/Ni/Sn samples with varying Ni thickness after reflow at 250 °C for 1 min immediately after electrodeposition. Three different Ni thicknesses used in this study are 35 nm, 70 nm and 150 nm. From this point onward, samples with Ni thickness of 35 nm, 70 nm and 150 nm will be designated as Cu/Ni-35/Sn, Cu/Ni-70/Sn, and Cu/Ni-150/Sn, respectively. Figure 3a–d shows cross-sectional FESEM micrographs of Cu/Sn, Cu/Ni-35/Sn, Cu/Ni-70/Sn, and Cu/Ni-150/Sn samples before reflow. The identities of the layers were determined by EDX analysis. The nature of the IMC was found out from the reaction of elements present. In Figure 3a–d, the layers with darker contrast are Cu while that with lighter contrast are Sn. Within the light grey Sn layers, a darker grey phase identified as Cu_6_Sn_5_ is seen in Cu/Ni/Sn sample (Figure 3a). As for samples with Ni insertion in between Cu and Sn, no IMC formation is seen. The presence of Ni layers was confirmed with EDX line scans profiles [15]. It may be noted that these samples were stored at room temperature for 5 days prior to taking the micrographs.

Figure 3e–h presents micrographs of Cu/Sn, Cu/Ni-35/Sn, Cu/Ni-70/Sn and Cu/Ni-150/Sn after reflow. In the reflowed Cu/Sn sample (Figure 3e), layers with the darkest contrast correspond to the Cu layer while layers with lightest contrast correspond to Cu_6_Sn_5_ phase. The continuous darker grey layers in between Cu and Cu_6_Sn_5_ interface and the discontinuous layers in the middle of the Cu_6_Sn_5_ IMC correspond to the Cu_3_Sn phase (Figure 3e). Discontinuous Cu3Sn IMC formed in the middle of the Cu_6_Sn_5_ phase due to reaction of the middle layer of copper with its surrounding tin during reflow. As for Figure 3f–h, layers with darker contrast represents Cu while lighter contrast represents the (Cu,Ni)_6_Sn_5_ phase. With the addition of Ni, it is seen that (Cu,Ni)_6_Sn_5_ IMC was formed regardless of the amount of Ni added. Cu/Ni-35/Sn has been converted completely into (Cu,Ni)_6_Sn_5_ (Figure 3f). However, for Cu/Ni-70/Sn and Cu/Ni-150/Sn samples (Figure 3g,h), unreacted copper (darker contrast) in between (Cu,Ni)_6_Sn_5_ (lighter contrast), is still left after reflow. The thickness of the unreacted copper increased with increasing Ni thickness. This suggests that Ni starts acting as a barrier layer between Cu and Sn when its thickness increased to 70 nm.

#### 2.2.2. Long Reflow

**Figure 4:**
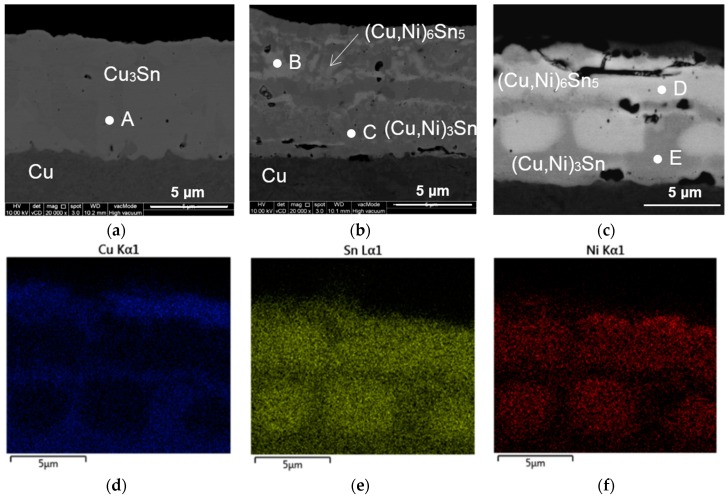
FESEM micrographs showing cross sectional images of (**a**) Cu/Sn; (**b**) Cu/Ni-35/Sn and (**c**) Cu/Ni-70/Sn and EDX elemental maps of (**d**) Cu; (**e**) Sn; and (**f**) Ni, of Cu/Ni-70/Sn after reflow at 300 °C for 60 min.

In order to further understand the growth of Cu-Sn IMC, longer reflow was done for 60 min at 300 °C. Figure 4 shows the FESEM cross-sectional images of Cu/Sn, Cu/Ni-35/Sn, and Cu/Ni-70/Sn after long reflow. Table 2 shows the EDX composition of the IMCs at different spots designated by A–E in Figure 4. In Figure 4a, it is seen that in the Cu/Sn system, all Cu and Sn layers have been transformed into a uniform layer of Cu_3_Sn. But, with the insertion of 35 nm of Ni, a lighter grey-coloured ribbon-like layer of (Cu,Ni)_6_Sn_5_ IMC was found to be mixed with (Cu,Ni)_3_Sn IMC which has a darker grey contrast. An increase of Ni thickness to 70 nm results in an increase in the amount of (Cu,Ni)_6_Sn_5_ (lighter contrast). This sample (Figure 4b) shows a brick-and-mortar type structure with bricks of (Cu,Ni)_6_Sn_5_ surrounded by (Cu,Ni)_3_Sn. Figure 4d–f shows EDX elemental maps of the Cu/Ni-70/Sn sample. Ni, which is indicated by the colour red is observed to segregate more to the light grey colour “brick” region (Figure 4c,f). EDX results in Table 1 shows that the light grey region is (Cu,Ni)_6_Sn_5_. Thus, Ni is seen to have a tendency to segregate to the (Cu,Ni)_6_Sn_5_ phase. Higher solubility limit of Ni in Cu_6_Sn_5_ compared with Cu_3_Sn is suggested to be the reason for the segregation [16].

**Figure 5:**
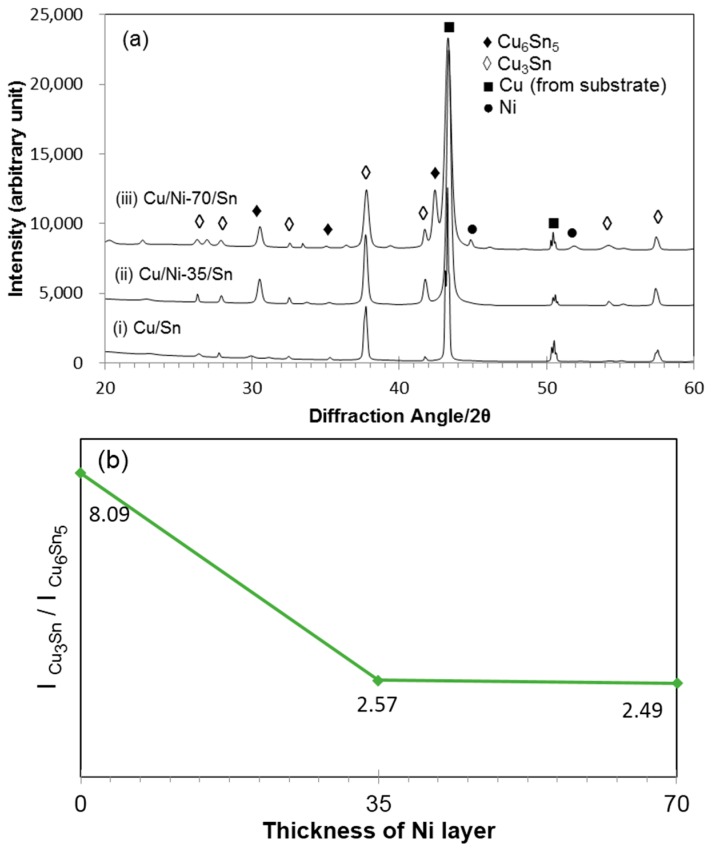
(**a**) XRD patterns for (i) Cu/Sn; (ii) Cu/Ni-35/Sn and (iii) Cu/Ni-70/Sn after reflowing at 300 °C for 60 min and (**b**) ratio of Cu_3_Sn peak intensity to that of Cu_6_Sn_5_ peak intensity for Cu/Sn, Cu/Ni-35/Sn, and Cu/Ni-70/Sn after reflow at 300 °C for 60 min.

Figure 5a shows the XRD patterns for Cu/Sn, Cu/Ni-35/Sn and Cu/Ni-70/Sn after reflow at 300 °C for 60 min. In all cases, Cu peaks from the substrate are observed at 43.41° and 50.55° which correspond to the Cu (111) and Cu (002) planes. Cu_6_Sn_5_ peaks are seen at 30.11° and 35.16° which correspond to the (311) and (40$\bar{2}$) planes. Cu_3_Sn peaks are seen at 27.99°, 32.66°, 37.89°, 41.99°, and 57.83° which corresponds to the (011), (110), (020), (002), and (022) planes, respectively. With the addition of 35 nm of Ni, it can be seen that there is an increase in the intensity of the Cu_6_Sn_5_ IMC peak at the diffraction angle of 30.11°. With a further increase of Ni to 70 nm, XRD patterns show Ni peaks at 44.34° and 51.66° corresponding to the (111) and (200) planes. The existence of these Ni peaks suggests that 70 nm of Ni layers are still not completely consumed after 60 min of reflow at 300 °C.

Figure 5b shows the ratio of the peak intensities for Cu_3_Sn and Cu_6_Sn_5_ as a function of Ni layer thickness. The Cu_6_Sn_5_ and Cu_3_Sn peaks used for the calculation are 30.11° and 37.89°, respectively, which are the most prominent peaks for each phase in the XRD pattern. From Figure 5b, it is seen that the intensity ratio decreases which indicates that the amount of Cu_3_Sn formed decreases when the Ni thickness increased. With increasing Ni in the Cu/Sn system, more Ni atoms substituted the Cu atoms to form more (Cu,Ni)_6_Sn_5_ which are more stable than Cu_6_Sn_5_ IMC resulting in less transformation from (Cu,Ni)_6_Sn_5_ IMC to (Cu,Ni)_3_Sn IMC [17,18].

**Figure 6:**
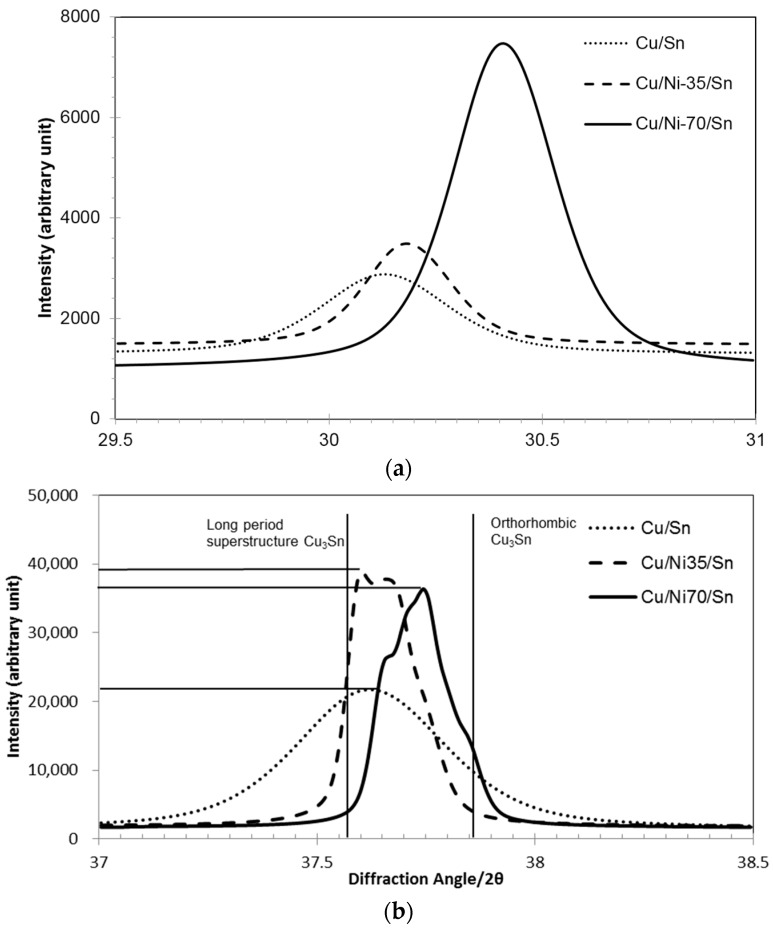
XRD patterns for Cu/Sn, Cu/Ni-35/Sn and Cu/Ni-70/Sn with diffraction angle of (**a**) 2θ = 29.5°–31° for the Cu_6_Sn_5_ (311) plane and (**b**) 2θ = 37°–38.5° for the Cu_3_Sn phase.

Figure 6 shows zoomed-in section of the XRD patterns of samples shown in Figure 5a recorded 29.5°–31° and 37°–38.5° recorded at a slow scan speed. This range of diffraction angles were selected to investigate possible peak shifts of the most prominent peaks of Cu_6_Sn_5_ and Cu_3_Sn IMC. It is seen in Figure 6a that the peak has shifted to the right when Ni atoms substitute Cu atoms in the (Cu,Ni)_6_Sn_5_ IMC. This indicates that the (311) interplanar spacing decreases upon Ni addition. This is in good agreement to previous study where it was found that Ni addition to Cu_6_Sn_5_ also decreased the lattice parameter [19]. As Ni atom substitutes Cu sites, the unit cell volume shrinks and the distance between the atoms shortens.

Cu_3_Sn possesses two features, orthorhombic unit cell, and a long period superstructure. The most prominent peak for the long period superstructure of Cu_3_Sn is at 37.56° for the (2 10 0) plane and that for the orthorhombic structure at 37.84° for the (021) plane are indicated by vertical lines. In the recorded patterns (Figure 6b), Cu_3_Sn in the Cu/Sn sample shows a peak which is close to the (2 10 0) long period superlattice peak. For the Cu_3_Sn IMC in the Cu/Ni/Sn sample, the peak tends to split into multiple sub-peaks and shift to higher angles. The peak for Cu/Ni-35/Sn sample is closer to that for Cu/Sn sample and exhibits two sub-peaks. The peaks for Cu/Ni-70/Sn shift closer to (021) Cu_3_Sn orthorhombic structures. These changes suggest that Ni atoms enter into the Cu_3_Sn lattice and exert an influence on the structure.

## 3. Discussion

### 3.1. IMC Formation during Solid State Reactions

During room temperature aging, copper and tin react to form Cu_6_Sn_5_ IMC even after one day of aging. In this present work, it is found that Cu_6_Sn_5_ IMC forms simultaneously at two places; (i) at the interface between Cu/Sn, possibly through rapid interstitial diffusion of Cu into bulk Sn and (ii) along the Sn grain boundaries.

Cu_6_Sn_5_ formation at the Cu/Sn interface was not observed in the earlier study done by Sobiech and co-workers [14]. One of the reasons for this might be related to the use of a lower resolution microscopy in that study. The formation of this thin layer of Cu_6_Sn_5_ at the entire Cu/Sn interface found in this study might be related to the fine copper microstructure. In this study, the copper layer was electrodeposited from alkaline pyrophosphate bath which is known to yield fine grains [20]. This is confirmed in the present study as the copper layers have grains in the range of 100–200 nm. These tiny grains also contain nano-twins as seen in Figure 2b. These finer microstructures can result in favourable nucleation sites for Cu_6_Sn_5_. In previous studies [12,13], deposition of Cu and Sn was done by thermal evaporation or electron beam evaporation which might yield a different structure and, thus, might provide fewer nucleation points.

Apart from the formation of Cu_6_Sn_5_ at the Cu/Sn interface, Cu also diffuses along Sn grain boundaries resulting in a slender Cu_6_Sn_5_ morphology at the grain boundaries (Figure 1a). Similar observations involving Cu diffusing along Sn grain boundaries were reported by Sobiech and co-workers [14,21]. After a complete coverage of Cu_6_Sn_5_ at Sn grain boundaries and the Cu/Sn interfaces, it grows by a volume diffusion of Cu into Sn in a direction perpendicular to Sn grain boundaries. This is how the Cu_6_Sn_5_ IMC grows wider and thicker after 24 days of room temperature aging.

**Figure 7:**
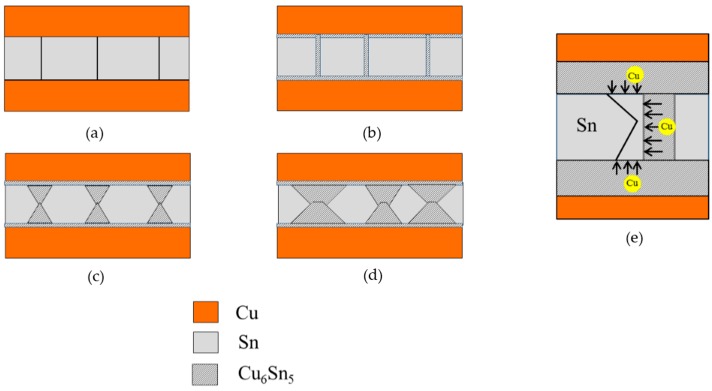
(**a**–**d**) Schematic diagram of Cu_6_Sn_5_ IMC formation in the Cu/Sn system during room temperature aging and (**e**) diffusion of Cu atoms into Sn to form Cu_6_Sn_5_ IMC.

A schematic of the stages in the formation of Cu_6_Sn_5_ IMC in the Cu/Sn system during room temperature aging is shown in Figure 7. The stages involved are (i) formation of a thin layer of Cu_6_Sn_5_ in between Cu and Sn and along Sn grain boundaries; (ii) growth of IMC along the Sn grain boundaries and (iii) volume diffusion of Cu into Sn. The unique “hourglass” shape of the Cu_6_Sn_5_ IMC (Figure 2b) formed at the grain boundaries is due to the diffusion of Cu from two direction as shown in Figure 7e. The corners situated at the junctions between substrate and grain boundaries receive Cu flux from two directions: (i) from Cu substrate side and (ii) from grain boundary side (Figure 7e). As a result, Cu_6_Sn_5_ grows faster here leading to the hourglass shape.

In the case when Ni is introduced between Cu and Sn (Figure 1 and Figure 2), the extent of IMC formation is less. It is seen in Figure 1 that Cu diffusion through the grain boundaries is restricted. Bader *et al.* has reported on a slower dissolution rate of Ni into Sn solders in comparison to Cu, and thus this can results in less IMC formation in the Ni-containing system [22]. It has been reported that the Cu diffusivity in Sn is 2.5 × 10^−7^ cm^2^/s [23] while Ni diffusivity in Sn is 5.4 × 10^−9^ cm^2^/s at a temperature above 160 °C [24]. Thus, it is expected that due to the slower diffusivity of Ni, Ni tends to block the diffusion path of Cu at the Sn grain boundaries which leads to less IMC formation.

Under room temperature aging conditions, it is observed that nanometric voids formed even after one day aging. With the increase of aging time to 24 days, the voids at the interface between Cu and Cu_6_Sn_5_ become prominent (Figure 2b). It has been reported that the voids can form in the Cu/Sn system due to Kirkendall effects. Kirkendall voids are reported to form at the Cu/Cu_3_Sn interface during solid state reactions because of the large difference in diffusion fluxes of Cu and Sn [12,25]. In this study, voids formed at the Cu/Cu_6_Sn_5_ interface due to the faster Cu diffusion leaving Cu vacancies at the Cu/Cu_6_Sn_5_ interface. However, when Ni is introduced into the Cu/Sn system, it is observed that the number of voids is less and the size of the voids is smaller. One reason for this might be the higher Ni diffusion flux in the system which can lead to a lesser difference in diffusion flux of Cu and Ni *versus* Sn [26].

### 3.2. IMC Formation during Solid Liquid State Reactions

#### 3.2.1. Dissolution of Ni during Reflow

From Figure 3, it is shown that only (Cu,Ni)_6_Sn_5_ IMC was formed after reflow regardless of the thickness of the Ni layer inserted between Cu and Sn layers. This is expected as the amount of Ni inserted into the Cu/Sn system is too low, 2 at %–8 at %, which is calculated from the deposited thickness of the layers. With this amount of Ni in the system, Ni_3_Sn_4_, and Ni_3_Sn_2_ IMC do not form [27]. The amount of Ni introduced is only sufficient for Ni to substitute Cu in the (Cu,Ni)_6_Sn_5_ IMC. However, when the thickness of Ni increases, the amount of unreacted copper increased (Figure 3f–h). It has been reported earlier [3,28] that some metallic nanoparticles including Ni undergo reactive dissolution during reflow. Thus, at smaller thickness, e.g., 35 nm, most of the Ni is expected to dissolve and react to form IMC. At higher thickness e.g., 70 nm, only a part of Ni layer is thought to have been dissolved. The remaining portion of the Ni layer acts as a barrier film. It has been reported that the diffusivity of Cu into Ni is very slow, 2.2 × 10^−18^ cm^2^/s at 300 °C [29]. Thus, when the thickness of Ni increased, the diffusion of Cu passing through Ni layers to reach the Sn is slower. This results in more unreacted copper when the thickness of Ni in the system increased.

#### 3.2.2. Effect of Ni Addition in Cu_6_Sn_5_ IMC

**Figure 8:**
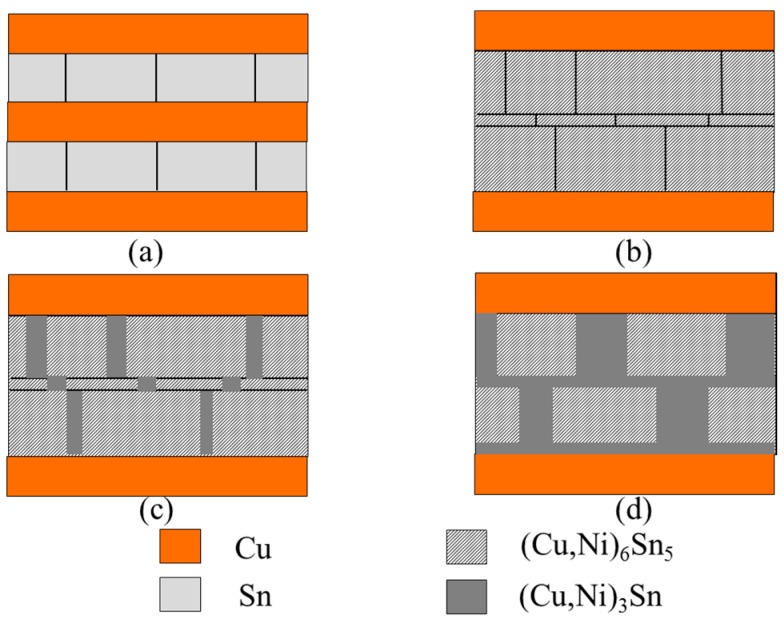
Schematic of the stage of growth of IMCs after long reflow at 300 °C for 60 min.

From the elemental maps in Figure 4, it is observed that Ni preferentially segregates to the (Cu,Ni)_6_Sn_5_ phase. EDX results for the (Cu,Ni)_6_Sn_5_ formed in the Cu/Ni-70/Sn sample shows that the Ni content in the IMC is 17.85 at %. This is in agreement with results published previously where it was reported that the solubility of Ni in (Cu,Ni)_6_Sn_5_ was around 29 at % [4]. The brick-wall morphology structure seen in Figure 4 is suggested to result from the growth of the Cu_3_Sn phase happening along the grain boundaries of the (Cu,Ni)_6_Sn_5_ IMC. Schematic in Figure 8 suggests steps in the growth of (Cu,Ni)_3_Sn during long reflow. It will be worthwhile to investigate the effect of the brick-wall morphology of the composite, (Cu,Ni)_6_Sn_5_ + (Cu,Ni)_3_Sn on mechanical properties and crack propagation mechanisms.

Peak intensity from XRD patterns (Figure 6) shows an increase for Cu_6_Sn_5_ and a decrease for Cu_3_Sn IMC as the thickness of Ni in the sample increases from 35 nm to 70 nm. This shows that Ni addition to the (Cu,Ni)_6_Sn_5_ IMC stabilises the phase and, thus, there is less transformation to (Cu,Ni)_3_Sn IMC. This is in good agreement to studies done by Yu [18] and Nogita [19]. Nogita reported that 9 at % of Ni in the (Cu,Ni)_6_Sn_5_ IMC is sufficient to stabilise the IMC in the hexagonal structure. Yu *et al.* reported that when Ni substituted Cu atoms to form (Cu,Ni)_6_Sn_5_, volume shrinkage happens and the distance between Cu and Sn atoms is shortened. This resulted in much stronger bonds and, thus, a more stable phase.

#### 3.2.3. Substitution of Ni Atoms in Cu Lattice in Cu_3_Sn IMC

(Cu,Ni)_3_Sn formed in the Cu/Ni-35/Sn and Cu/Ni-70/Sn samples after long reflow. Though this IMC is rarely reported in solder joints, literature shows that Ni can substitute Cu in Cu_3_Sn to become (Cu,Ni)_3_Sn in bulk sample prepared by melting and term aging [16,18,30,31]. Table 3 shows the effect of concentration and heating parameters on Ni concentration in (Cu,Ni)_3_Sn IMC. Oberndoff [31] has reported on the formation of (Cu,Ni)_3_Sn after samples containing 40 at % Sn, 35–50 at % Cu and 10–25 at % Ni after annealing at 235 °C for 1100–1700 h. Oberndoff also reported that the solubility of Ni in (Cu,Ni)_3_Sn can be as high as 3 at %. Lin and co-workers [16] also observed the formation of (Cu,Ni)_3_Sn when annealing was done at 240 °C for 1695 h on two groups of samples, with compositions (i) 25 at % Sn, 5–70 at % Cu, 5–70 at % Ni and (ii) 40 at % Sn, 30–50 at % Cu, 10–30 at % Ni. Lin *et al.* reported that when the concentration of Sn was low (25 at %), the amount of Ni in (Cu,Ni)_3_Sn varied from 5 at % to 70.8 at %. However, when the concentration of Sn was high (40 at %), the amount of Ni in (Cu,Ni)_3_Sn only ranged from 1.5 at % to 4.3 at %. In the present study, EDX results show that the amount of Ni in the (Cu,Ni)_3_Sn is 1.87 at % for the Cu/Ni-35/Sn sample and 4.56 at % for the Cu/Ni-70/Sn sample. This is in good agreement with previous studies as the concentration of Sn used in this study is around 40–50 at %. The formation of (Cu,Ni)_3_Sn IMC found in this study occurs because of the limited Sn supply in the samples used. Past studies used solder balls, solder pastes, or thick layers of Sn solders which can provide an effectively unlimited supply of Sn for the reactions of Cu-Sn IMCs. When the supply of Sn is limited, the amount of Cu_6_Sn_5_ IMC formed is limited as well. Since Ni only has a solubility of around 27 at % in (Cu,Ni)_6_Sn_5_, Ni atoms will also substitute Cu in the (Cu,Ni)_3_Sn IMC as the reaction is thermodynamically possible [18].

From the XRD patterns (Y 6), it is seen that peaks for the Cu_3_Sn phase has shifted to higher angles when Ni substitutes Cu. Substitution of Cu by Zn was studied by van Sande *et al.* [32]. Addition of Zn to Cu_3_Sn was found to reduce the long period in the superstructure. The periodicity was also reduced when Ni was added [33]. Further studies are necessary to understand in detail the effect of Ni addition on the structure of Cu_3_Sn.

## 4. Materials and Methods

In this study, the multilayer interconnect systems were prepared on commercially pure copper substrates by electrodeposition. Sequential deposition was carried out to prepare both Cu/Sn and Cu/Ni/Sn multilayer interconnects. Cu/Sn multilayers were prepared in the sequence of Cu/Sn/Cu/Sn/Cu while Cu/Ni/Sn multilayers were prepared in the sequence of Cu/Ni/Sn/Ni/Cu/Ni/Sn/Ni/Cu. For the sake of simplicity, Cu/Sn/Cu/Sn/Cu and Cu/Ni/Sn/Ni/Cu/Ni/Sn/Ni/Cu will be designated by Cu/Sn and Cu/Ni/Sn, respectively. Copper deposition in this study, was performed using the alkaline pyrophosphate copper bath (CuSO_4_·5H_2_O, 30 g/L; Na_4_PO_2_O_7_, 120 g/L; NH_3_, 1 mL/L). The bath pH was adjusted to 8.5 by using sulphuric acid for the pyrophosphate copper bath. Watts bath (NiSO_4_, 150 g/L; NiCl_2_, 60 g/L; H_3_BO_3_, 37.5 g/L) was used for the deposition of nickel. Methanesulfonic acid (MSA)-based bath (SnSO_4_, 30 g/L; gelatin, 1 g/L; hydroquinone, 5 g/L; MSA, 120 mL/L) was used for the deposition of tin [15,34]. Stirring condition was set at 80 rpm for all baths. Deposition current density was 10 mA/cm^2^ for copper deposition and 20 mA/cm^2^ for tin and nickel deposition. The deposition time required to achieve required thickness was estimated using Faraday’s law. Copper was plated to achieve a thickness of 1000 nm while tin was plated to a thickness of 2000 nm. Nickel was plated to a thickness of 35 nm, 70 nm, and 150 nm to study the effect of varying Ni concentration in the Cu/Sn system.

The multi-layered samples were subjected to different processing conditions to study their intermixing and phase transformation characteristics: (i) room temperature aging; (ii) short reflow and (iii) long reflow. Room temperature aging studies were done at 25 °C. Short reflow was done in a FT-02 convection reflow oven at 250 °C while long reflow was done in a tube furnace in 2% H_2_/N_2_ atmosphere at 300 °C. FEI Quanta 450 (Houston, TX, USA) Field-Emission Scanning Electron Microscope (FESEM) was used to examine the cross-section of the electrodeposits. The number and size of the voids formed in the Cu/Sn and Cu/Ni/Sn samples were calculated from four micrographs for each condition. Energy dispersive X-ray spectroscopy (EDX, Oxford Instruments, Oxfordshire, UK) was used to determine the composition of the multilayer system. FIB milling was done in a FEI Helios Nanolab 650 Dual Beam FESEM. The phases of the multilayer sample after long reflow were investigated by X-ray Diffraction (PANalytical, Almelo, The Netherlands) using a PanAnalytical diffractometer with Cu Kα radiation which has a wavelength of 1.5418 Å. The step size used was 0.26° and the scan step time was 2.11 s. For slower scans, a step size of 0.01° and scan step time of 8.80 s were used. Peaks shown in the XRD pattern were identified by using the Powder Diffraction File card (JCPDS).

## 5. Conclusions

1. In the electrodeposited Cu/Sn system, Cu_6_Sn_5_ IMC is seen to grow even after one day of room temperature aging, while less Cu-Sn IMC is found in Cu/Ni/Sn system under the same conditions. This finding is important to prevent premature intermixing between the multilayers prior to reflowing during manufacturing process.
2. During solid state reaction in the Cu/Sn system, the growth of the Cu_6_Sn_5_ first started through rapid reaction at the Cu/Sn interface followed by grain boundary diffusion of Cu atoms into the Sn grain boundaries. With the addition of Ni in between Cu and Sn, Ni atoms are suggested to block the diffusion path of Cu atoms into Sn grain boundaries and slow down IMC formation.
3. During liquid solid reaction, at 70 nm thickness Ni dissolution is incomplete in the Cu/Ni/Sn multilayers. Thus, to achieve homogeneous IMC layer during short reflow, Ni thickness less than 70 nm is recommended. The addition of Ni suppresses formation of Cu_3_Sn IMC, regardless of the thickness of the Ni layers.
4. After 60 min of long reflow, Cu/Sn multilayers have been transformed totally into Cu_3_Sn. In the Cu/Ni/Sn system, Ni atoms take part in the formation of (Cu,Ni)_6_Sn_5_ and, thus, stabilizes the IMC and retards transformation into (Cu,Ni)_3_Sn. Formation of (Cu,Ni)_3_Sn is suspected to be due to the limited Sn supply in the system.
5. The mechanical properties of the unique “brick-wall” morphology of the [(Cu,Ni)_6_Sn_5_ + (Cu,Ni)_3_Sn] composite formed in the Cu/Ni-70/Sn samples after long reflow would be interesting, as it may influence fracture propagation.

## Figures and Tables

**Table 1:** Number of voids *vs.* diameter of voids in Cu/Sn and Cu/Ni/Sn aged at room temperature for 24 days.

Diameter of Voids (nm)	Number of Voids			
	CuSn		CuNiSn	
	1 Day	24 Days	1 Day	24 Days
<10	35	35	31	88
11–20	112	137	110	114
21–30	31	22	11	6
31–40	9	17	5	5
41–50	0	27	4	6
>50	1	26	5	10

**Table 2:** Number of voids *vs.* diameter of voids in Cu/Sn and Cu/Ni/Sn aged at room temperature for 24 days.

Spot	Composition (at %)			Ratio	Phase Identified
	Cu	Sn	Ni		
A	76.27	23.73	–	Cu:Sn 3.21:1.00	Cu_3_Sn
B	55.27	35.12	9.61	(Cu + Ni):Sn 9.28:5.00	(Cu,Ni)_6_Sn_5_
C	72.35	25.78	1.87	(Cu + Ni):Sn 2.88:1.00	(Cu,Ni)_3_Sn
D	37.74	44.41	17.85	(Cu + Ni):Sn 6.26:5.00	(Cu,Ni)_6_Sn_5_
E	70.06	25.38	4.56	(Cu + Ni):Sn 2.94:1.00	(Cu,Ni)_3_Sn

**Table 3:** Effects of Concentration and Heating Parameters on Ni concentration in (Cu,Ni)_3_Sn IMC.

Concentration (at %)			Temperature (°C)	Duration (h)	Ni Concentration in (Cu,Ni)_3_Sn (at %)	Reference
Sn	Cu	Ni				
40	35–50	10–25	235	1100–1700	3	[31]
25	5–70	5–70	240	1695	5–70.8	[16]
40	30–50	10–30	240	1695	1.5–4.3	[16]
